# A causal modelling framework for reference-based imputation and tipping point analysis in clinical trials with quantitative outcome

**DOI:** 10.1080/10543406.2019.1684308

**Published:** 2019-11-12

**Authors:** Ian R. White, Royes Joseph, Nicky Best

**Affiliations:** a MRC Biostatistics Unit, Cambridge, UK; b MRC Clinical Trials Unit at UCL, London, UK; c R&D Biostatistics, GlaxoSmithKline, Uxbridge, UK

**Keywords:** Clinical trial, de facto estimand, missing data, Multiple imputation, Sensitivity analysis, Reference-based imputation, Causal inference

## Abstract

We consider estimation in a randomised placebo-controlled or standard-of-care-controlled drug trial with quantitative outcome, where participants who discontinue an investigational treatment are not followed up thereafter, and the estimand follows a treatment policy strategy for handling treatment discontinuation. Our approach is also useful in situations where participants take rescue medication or a subsequent line of therapy and the estimand follows a hypothetical strategy to estimate the effect of initially randomised treatment in the absence of rescue or other active treatment. Carpenter et al proposed reference-based imputation methods which use a reference arm to inform the distribution of post-discontinuation outcomes and hence to inform an imputation model. However, the reference-based imputation methods were not formally justified. We present a causal model which makes an explicit assumption in a potential outcomes framework about the maintained causal effect of treatment after discontinuation. We use mathematical argument and a simulation study to show that the “jump to reference”, “copy reference” and “copy increments in reference” reference-based imputation methods, with the control arm as the reference arm, are special cases of the causal model with specific assumptions about the causal treatment effect. We also show that the causal model provides a flexible and transparent framework for a tipping point sensitivity analysis in which we vary the assumptions made about the causal effect of discontinued treatment. We illustrate the approach with data from two longitudinal clinical trials.

## 1. Introduction

Missing outcome data represent a major threat to the validity of randomised controlled trials (RCTs), and appropriate analysis methods have been much discussed. An influential report showed that different analysis methods may target different estimands (different measures of treatment effect) and argued that specification of the estimand is an important part of trial design and should inform trial analysis and reporting (National Research Council 2010). Regulators have joined the call for estimands to be defined clearly, and the International Council for Harmonisation of Technical Requirements for Pharmaceuticals for Human Use (ICH) Steering Committee has endorsed the development of new regulatory guidance on the choice of estimands and sensitivity analysis in clinical trials (European Medicines Agency 2017).

We consider two types of estimand considered by the National Research Council (2010): (E1) difference in outcome improvement at the planned endpoint if all participants had tolerated or adhered to trial protocol; (E2) difference in outcome improvement at the planned endpoint for all randomised participants. The former measures how treatment works in an ideal setting (efficacy), while the latter measures how treatment might work in practice (effectiveness). To encompass outcomes that measure harms of treatment, Carpenter et al. (2013) (henceforth CRK) proposed the broader terms *de jure* and *de facto* estimand for (E1) and (E2) respectively. The ICH E9(R1) draft addendum (European Medicines Agency 2017) refers to a “hypothetical strategy” and a “treatment policy strategy” in defining estimands (E1) and (E2) respectively.

Sometimes investigators continue to collect data after treatment discontinuation. The use of such *off-treatment* data depends on the estimand (Permutt 2016). For the estimation of a *de jure* estimand, off-treatment data for participants who discontinued randomised treatment could be used in a complier average causal effect analysis (Dunn et al. 2003). In practice, however, off-treatment data are typically either not collected or excluded from the primary analysis, and the missing data are assumed to be missing at random (MAR): that is, it is assumed that participants who discontinued treatment would have benefited from continued treatment in the same way as those who remained on treatment. However, estimation of a *de facto* estimand ideally makes use of the off-treatment data, which should be collected where possible (National Research Council 2010). When all discontinuers are followed up and complete outcome data are obtained, the *de facto* estimand can be directly estimated by comparing observed means (Little and Kang 2015).

This paper considers estimation of a *de facto* estimand for a quantitative outcome when off-treatment data are not collected. For participants who have discontinued treatment, this requires assumptions about whether and to what extent they continue to benefit from their previous treatment. Our approach is also relevant to the situation in which rescue treatment (over and above the per-protocol treatment regime for the control arm) is available for those who discontinue randomised treatment, but interest is in the effect attributable to the initially randomised treatment without the confounding effects of rescue medications (corresponding to estimand 6 in Mallinckrodt et al. (2012)), and data after rescue are either unavailable or are ignored.

In the previous work on this topic, Little and Yau (1996) presented a multiple imputation (MI) approach that could incorporate a variety of alternative assumptions about the treatment effect after treatment discontinuation for the estimation of *de facto* estimands in RCTs. CRK presented a generic algorithm for MI of post-discontinuation outcome data. They assumed that post-discontinuation outcomes in a given trial arm behave in some way like outcomes in a reference arm (often the control arm), and proposed various specific methods for forming the imputation distribution. These methods have been called “reference-based imputation” (RBI) or “control-based imputation” methods. However, CRK did not theoretically justify these methods. In this paper, we assume that participants who have discontinued their randomised treatment receive treatment that is similar to that allocated to the control arm: thus for reference-based imputation, we take the reference to be the control treatment, where this is typically either placebo or standard of care.

Specification of estimands is clarified by using counterfactual outcomes – outcomes that have not been or could not have been observed. Such counterfactuals are best described using potential outcomes notation (Angrist et al. 1996; Little and Rubin 2000). Our aims in this paper are first to propose and implement a causal model, using explicit assumptions about the causal effect of a previously discontinued treatment, and second to show that three of the RBI methods are special cases of the causal model, and hence to provide their theoretical justification.

Implementing the causal model requires untestable assumptions, so we need sensitivity analyses to understand the impact of these assumptions on inferences and conclusions from the primary analysis. Kenward et al. (2001) described a principled approach to sensitivity analyses which varies a sensitivity parameter that quantifies deviations from the missing data assumption. A tipping point sensitivity analysis (e.g. Yan et al. (2009); Liublinska and Rubin (2014)) extends this approach by varying the sensitivity parameter until the conclusion from the primary analysis is overturned. The third aim of this paper is to propose a tipping point sensitivity analysis using the causal model.

The paper is organised as follows: In Section 2, we set out notation and define the RBI methods. Section 3 contains the key new material: here we set out the causal model and discuss equivalence with RBI methods. In Section 4, we discuss implementation. In Section 5 we verify the equivalence of RBI and causal model estimates in a simulation study. In Section 6, we illustrate the proposed approach and demonstrate the tipping point analysis with two example data sets. We conclude with summary remarks in Section 7.

## 2. Reference-based imputation (RBI) methods

### 2.1. Notation

We consider a two-arm longitudinal RCT with quantitative outcome observations scheduled at baseline and at $t_{max}$ occasions after randomisation. Let Z be the random variable for the participant’s randomised treatment arm: Z=a for the active treatment arm and Z=c for the control arm. Let $Y_{t}$ be the random variable for the participant’s outcome at visit $t=0,...,t_{max}$. It is convenient to define $Y_{\leq t}=(Y_{0},\ldots ,Y_{t})$, the vector of all outcomes up to and including visit t; $Y_{>t}=(Y_{t+1},\ldots ,{Y_{t}}_{max})$, the vector of all outcomes after visit t; and $Y=(Y_{0},\ldots ,{Y_{t}}_{max})$, the vector of all outcomes. Let D be the random variable for the participant’s last visit prior to discontinuing treatment, so $D=0,...,t_{max}$. $Y_{t}$ is observable for all t but only observed for t≤D, because we assume no off-treatment data. We aim to impute the unobserved values of $Y_{t}$ for t>D: we stress that these are the outcomes that existed but were unobserved, not the outcomes that would have existed if treatment had been continued.

We define the potential outcome $Y_{t}(s)$ at visit t as the outcome that would have been observable if, possibly contrary to fact, a participant received active treatment for s periods only. In particular, $Y_{t}(0)$ is the potential outcome if never treated, and $Y_{t}(t_{max})$ is the potential outcome if always treated. We define $Y_{\leq t}(s)$, $Y_{>t}(s)$ and Y(s) as before. We let $\mu_{t}(s)=\;E\;\left[Y_{t}(s)\right]$, the mean of the potential outcome at visit t if active treatment was received for s periods only. Similarly, we define ${\mu_{\leq }}_{t}(s)$, ${\mu_{>}}_{t}(s)$ and μ(s). The variance-covariance matrix of the potential outcomes is $\Sigma (s)=var\left(Y(s)\right)$ with submatrices $\Sigma_{\leq t\leq t}(s)$, $\Sigma_{>t>t}(s)$ and $\Sigma_{>t\leq t}(s)$. We define the matrix of regression coefficients of potential outcomes after visit t on those up to visit t as $\beta_{t}(s)=\Sigma_{>t\leq t}(s)\Sigma_{\leq t\leq t}(s{)}^{-1}$, and the residual variance of the potential outcomes after visit t given those up to visit t as $\Omega_{t}(s)=\Sigma_{>t\leq t}(s)\Sigma_{\leq t\leq t}(s{)}^{-1}\Sigma_{>t\leq t}(s{)}^{T}$.

**Figure 1.**
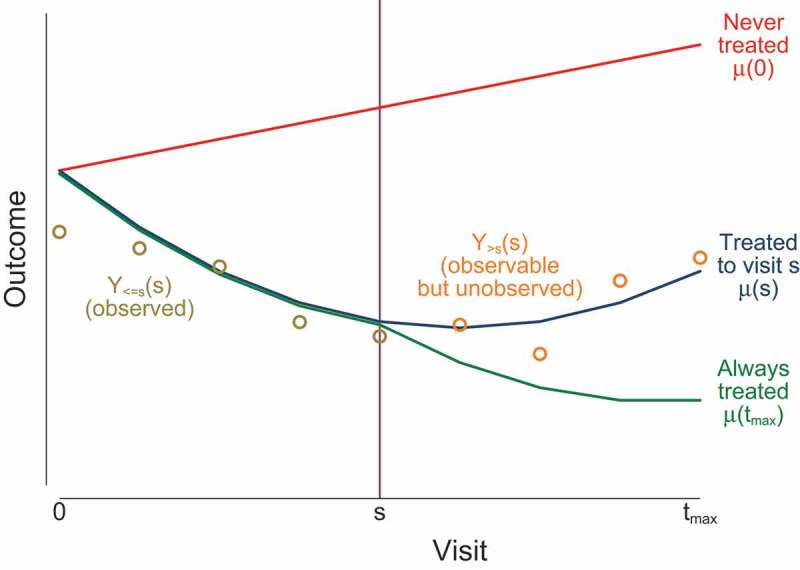
Notation illustrated. Lines indicate mean potential outcomes under three potential treatment scenarios. Circles indicate observable outcomes for a participant who discontinues treatment at visit s.

Figure 1 illustrates this notation in the case of an adverse outcome which deteriorates (increases) in the absence of treatment and improves (decreases) in the presence of treatment. If treatment is discontinued at time s then mean outcomes up to time s are unaffected ($\mu_{t}(s)=\mu_{t}(t_{max})$ for t≤s) but outcomes after time s are worsened ($\mu_{t}(s)>\mu_{t}(t_{max})$ for t>s). The notation is summarised in supplementary appendix A.

This potential outcomes notation allows for only one type of treatment. We assume that the observed outcomes are not affected by other treatments. For the outcomes after treatment discontinuation, we assume either that rescue treatment (over and above the per-protocol treatment regime for the control arm) is not available or that interest is in the effect attributable to the initially randomised treatment without the confounding effects of rescue medications.

The *de jure* estimand (estimand E1) at visit t>0 is $\;E\;\left[Y_{t}(t)-Y_{t}(0)\right]$. The *de facto* estimand (estimand E2) is the estimand of interest in this paper and is $\;E\;\left[Y_{t}(D)|Z=a\right]-\;E\;\left[Y_{t}(0)|Z=c\right]$. Often, primary interest is in the last visit, $t=t_{max}$.

### 2.2. Reference-based imputation

CRK proposed a generic MI algorithm for this setting:

1. For each treatment arm, fit a multivariate normal model to all observed data, using a Bayesian approach with an improper prior and assuming MAR. The model should have unstructured mean and unstructured variance-covariance matrix.
2. For each treatment arm, draw a mean vector and variance-covariance matrix from the posterior distribution.
3. For each treatment arm and each possible treatment discontinuation visit t, use the draws to build the hypothetical joint distribution of the outcomes $Y_{\leq t}$ up to time t and the outcomes $Y_{>t}$ after time t, using one of the methods described below.
4. For each treatment arm and each observed treatment discontinuation visit t, construct the imputation distribution of $Y_{>t}$ given $Y_{\leq t}$. Sample $Y_{>t}$ from this conditional distribution, to create a “completed” data set.
5. Repeat steps 2–4 m times, resulting in m imputed data sets.
6. Analyse each imputed data set and combine the results using Rubin’s rules (Rubin 1987).

To understand the assumptions behind the CRK algorithm, we express it using the potential outcomes notation.

In step 1, the model is fitted to each treatment arm separately. In the control arm, the observed outcomes are $Y_{t}=Y_{t}(0)$. In the active treatment arm, the observed outcomes are $Y_{t}=Y_{t}(t_{max})$, because we assume no off-treatment data. Hence, under MAR assumptions that we make explicit in Section 3.1, the multivariate normal model fitted to the control arm has mean μ(0) and variance Σ(0), and that fitted to the active treatment arm has mean $\mu (t_{max})$ and variance $\Sigma (t_{max})$.

In step 2, values of μ(0), Σ(0), $\mu (t_{max})$ and $\Sigma (t_{max})$ are drawn from their posterior distributions.

In step 3, the drawn values are used to build hypothetical joint distributions of Y. Specifically, for participants in the active treatment arm who discontinue treatment at time t, a joint distribution is built for Y|Z=a,D=t. CRK proposed using a multivariate normal distribution. Five methods are mainly distinguished by their choice of mean:

- Missing at random (MAR): mean = $\mu (t_{max})$.
- Last mean carried forward (LMCF): mean = $\left({\mu_{\leq }}_{t}(t_{max}),\mu_{t}(t_{max})e_{t_{max}-t}\right)$ where $e_{p}$ is a row vector (1,…,1) of length p.
- Copy reference (CR): mean = μ(0).
- Jump to reference (J2R): mean = $\left({\mu_{\leq }}_{t}(t_{max}),{\mu_{>}}_{t}(0)\right)$.
- Copy increments in reference (CIR):
  $$mean=\left({\mu_{\leq }}_{t}(t_{max}),{\mu_{>}}_{t}(0)+\{\mu_{t}(t_{max})-\mu_{t}(0)\}e_{t_{max}-t}\right).$$

CRK proposed corresponding variance matrices. We simplify their description by observing that only the regression coefficient matrix and conditional variance matrix of the potential outcomes after visit t given those up to visit t are used in later steps. CRK set these to be $\beta_{t}(t_{max})$ and $\Omega_{t}(t_{max})$ for MAR and LMCF, and $\beta_{t}(0)$ and $\Omega_{t}(0)$ for J2R, CIR and CR. An approach that we call *RBI alternative* instead uses $\beta_{t}(t_{max})$ and $\Omega_{t}(t_{max})$ for all RBI methods.

In step 4, the joint distributions above are used to derive conditional distributions for ${Y_{>}}_{t}(t)|Z=a,D=t,{Y_{\leq }}_{t}(t)$. Under J2R, for example, this is

$$N\left({\mu_{>}}_{t}(0)+\beta_{t}(0)\left\{{Y_{\leq }}_{t}(t_{max})-{\mu_{\leq }}_{t}(t_{max})\right\},\Omega_{t}(0)\right)\;\;\;\;\;\;\;\;\;\;\;\;\;\;(RBI)\;$$

$$N\left({\mu_{>}}_{t}(0)+\beta_{t}(t_{max})\left\{{Y_{\leq }}_{t}(t_{max})-{\mu_{\leq }}_{t}(t_{max})\right\},\Omega_{t}(t_{max})\right)\;\;\;\;\;(RBI\;alternative)\;$$

The rest of the CRK algorithm follows standard MI methods, using the conditional distribution as the imputation model.

The hypothetical joint distributions under each of these methods are written in the notation of this paper in supplementary appendix B. The corresponding imputation distributions for the Z=a,D=t subgroup for any $t<t_{max}$ are given under “Reference-based imputation methods” in Table 1. The imputation means are written as a selection term, reflecting how the D=t subgroup differs from other participants, plus a term linearly related to the treatment effect up to time t. This motivates our causal model in section 3, which relates causal treatment effects after D to those up to D.

**Table 1.** Imputation distribution of $Y_{>t}(t)$ for $t<t_{max}$ given randomisation Z=a, past $Y_{\leq t}$ and treatment discontinuation visit D=t, under various reference-based imputation methods with control arm as reference (Carpenter et al. 2013) and under the causal model. $C_{t}$ is a ‘carry-forward’ $\left(t_{max}-t)\times (t+1\right)$ matrix containing t columns of zeroes and a final column of ones, so that $C_{t}\mu_{\leq t}(t)$ is a column vector containing $t_{max}-t$ copies of $\mu_{t}(t)$.

	Imputation distribution	
Method	Mean	Variance
*Reference-based imputation methods*		
MAR	$\beta_{t}(t_{max})\left\{Y_{\leq t}-\mu_{\leq t}(t)\right\}+\mu_{>t}(t_{max})$	$\Omega_{t}(t_{max})$
LMCF	$\beta_{t}(t_{max})\left\{Y_{\leq t}-\mu_{\leq t}(t)\right\}+C_{t}\mu_{\leq t}(t)$	$\Omega_{t}(t_{max})$
J2R	$\beta_{t}(0)\left\{Y_{\leq t}-\mu_{\leq t}(t)\right\}+\mu_{>t}(0)$	$\Omega_{t}(0)$
CIR	$\beta_{t}(0)\left\{Y_{\leq t}-\mu_{\leq t}(t)\right\}+C_{t}\left\{\mu_{\leq t}(t)-\mu_{\leq t}(0)\right\}\;\;+\mu_{>t}(0)$	$\Omega_{t}(0)$
CR*	$\beta_{t}(0)\left\{Y_{\leq t}-\mu_{\leq t}(t)\right\}\;+\beta_{t}(0)\left\{\mu_{\leq t}(t)-\mu_{\leq t}(0)\right\}+\mu_{>t}(0)$	$\Omega_{t}(0)$
*Causal model*		
	$\beta_{t}(t)\left\{Y_{\leq t}-\mu_{\leq t}(t)\right\}+K_{t}\left\{\mu_{\leq t}(t)-\mu_{\leq t}(0)\right\}\;+\mu_{>t}(0)$	$\Omega_{t}(t)$

* The CR mean is more simply written $\beta_{t}(0)\left\{Y_{\leq t}-\mu_{\leq t}(0)\right\}+\mu_{>t}(0)$, but the expression given here facilitates comparison with the other methods.

## 3. New causal model

In this section, we first set out the assumptions of the causal model, and then derive the imputation model.

### 3.1. Assumptions

**Assumption A1**. *Randomisation is independent of potential outcomes*: Z⊥⊥Y(s) *for all* s.

**Assumption A2**. (Y|Z=c) *is missing at random (MAR).*

Assumption A2 states that the observed outcomes in the control arm are MAR. If there are no missing data before treatment discontinuation, then we can also write this

$$p(D=t|Z=c,Y,D\geq t)=p(D=t|Z=c,Y_{\leq t},D\geq t)$$

for all t. In other words, treatment discontinuation in the control arm does not relate to future untreated outcomes, given the past and present.

**Assumption A3**. $\left(Y(t_{max})|Z=a\right)$ *is MAR.*

Assumption A3 states that the counterfactual fully treated outcomes in the active arm are MAR. If there are no missing data before treatment discontinuation, then we can also write this

$$p(D=t|Z=a,Y(t_{max}),D\geq t)=p(D=t|Z=a,Y_{\leq t}(t_{max}),D\geq t)$$

for all t. In other words, treatment discontinuation in the active arm does not relate to future counterfactual fully treated outcomes, given the past and present.

We do not assume that the actual outcomes in the active arm, (Y|Z=a), are MAR. Indeed, this is unlikely to be true, since (if treatment is effective) treatment discontinuation causally affects actual future outcomes. Thus, treatment discontinuation is allowed to relate to future *actual* outcomes, given the past and present.

**Assumption A4**. $Y_{>t}(t)|Y_{\leq t}(t)$ *follows a linear regression for each* t.

This assumption implies that the conditional mean of each future potential outcome $Y_{u}(t)$ (u>t) depends linearly on the past observed outcomes $Y_{1}(t),\ldots ,Y_{t}(t)$. We make no assumption of linearity in t or u, so that trajectories over time have no assumed form. A4 is true if Y(t) follows a multivariate Normal distribution. The linear regression has mean ${\mu_{>}}_{t}(t)+\beta_{t}(t)\{{Y_{\leq }}_{t}(t)-{\mu_{\leq }}_{t}(t)\}$ and residual variance matrix $\Omega_{t}(t)$.

**Assumption A5**. $p(D=t|Z=a,Y(t))=p(D=t|Z=a,Y_{\leq t}(t))$.

A5 states that treatment discontinuation at visit t is unaffected by future partly treated potential outcomes. It appears similar to the equation A3, but the latter refers instead to future *fully*-treated potential outcomes. If there are no missing data before treatment discontinuation then a stronger assumption which implies both A3 and A5 is

$$p(D=t|Z=a,Y(s),D\geq t)=p(D=t|Z=a,Y_{\leq t}(s),D\geq t)$$

for all t and all s>t.

**Assumption A6**. $\;E\;\left[Y_{>t}(t)-Y_{>t}(0)\right]=K_{t}\;E\;\left[Y_{\leq t}(t)-Y_{\leq t}(0)\right]$.

A6 is an explicit assumption about how the maintained effect of treatment after discontinuation relates to the effect of treatment before discontinuation. Equivalently,

(1) $$\mu_{>t}(t)-\mu_{>t}(0)=K_{t}\left\{\mu_{\leq t}(t)-\mu_{\leq t}(0)\right\}.$$

$K_{t}$ is a $\left(t_{max}-t)\times (t+1\right)$ matrix of sensitivity parameters: it is not identified by the data and must be specified by the user. Some suggestions for $K_{t}$ are made in Section 3.3.

Our model makes no assumption about how the effect of active treatment changes over time while active treatment is continued. However, implicit in assumption A6 is that there is no delayed response to the control treatment: thus when a patient discontinues randomised treatment, we assume the effects of any treatments they switch to are similar to the effects they would have experienced had they received the control treatment from the start of the trial.

### 3.2. Modelling outcomes after treatment discontinuation

In this subsection, we consider an individual in the active arm who stops treatment at visit $t<t_{max}$. We use the above assumptions to derive a model for this individual’s outcomes after treatment discontinuation, conditional on their history $Y_{\leq t}$. In section 3.3 we take this model as an imputation model and compare it with the RBI imputation models.

We write the conditional mean outcome after treatment discontinuation in this model as the sum of three terms:

(2) $$\begin{array}{rl} & E[Y_{>t}(t)|Z=a,Y_{\leq t},D=t]=\left\{E[Y_{>t}(t)|Z=a,Y_{\leq t},D=t]-\mu_{>t}(t)\right\}\\ & \;\;\;\;\;\;\;\;\;\;\;\;\;\;+\left\{\mu_{>t}(t)-\mu_{>t}(0)\right\}+\mu_{>t}(0) \end{array}$$

where the first term represents the difference between the subgroup who discontinue at visit t and the whole group (“selection term”), the second term represents the treatment effect in the whole group (“maintained treatment effect”), and the third term is the untreated mean.

We write the selection term as

(3) $$\begin{array}{cccc} \;E\;\left[Y_{>t}(t)|Z=a,Y_{\leq t}(t),D=t\right] & - & \mu_{>t}(t) & \\ \;=\;E\;\left[Y_{>t}(t)|Z=a,Y_{\leq t}(t)\right] & - & \mu_{>t}(t) & \;\;(by\;A5\;)\;\\ \;=\;E\;\left[Y_{>t}(t)|Y_{\leq t}(t)\right] & - & \mu_{>t}(t) & \;\;(by\;A1\;)\;\\ \;=\beta_{t}(t)\left\{Y_{\leq t}(t)-\mu_{\leq t}(t)\right\} & & & \;\;(by\;A4\;).\; \end{array}$$

Using assumption A6, and substituting (1) and (3) into (2) gives the mean of the imputation model:

(4) $$E[Y_{\text{ >}t}(t)|Z=a,Y_{\leq t},D=t]\;=\beta_{t}(t)\left\{Y_{\leq t}-\mu_{\leq t}(t)\right\}+K_{t}\left\{\mu_{\leq t}(t)-\mu_{\leq t}(0)\right\}+\mu_{\text{ >}t}(0).$$

For the variance, we approximate $var\left(Y_{>t}(t)|Z=a,Y_{\leq t}(t),D=t\right)$ by $var\left(Y_{>t}(t)|Z=a,Y_{\leq t}(t)\right)$ which is valid when differences between drop-out patterns are small compared with the variation in the data, and otherwise conservative. By A1, this is $var\left(Y_{>t}(t)|Y_{\leq t}(t)\right)=\Omega_{t}(t)$. This imputation distribution is given under “Causal model” in Table 1.

### 3.3. Using the causal model

We need to fix three parameters in order to identify the causal model: $K_{t}$, $\beta_{t}(t)$ and $\Omega_{t}(t)$. In most cases the post-discontinuation treatment effect may be assumed to depend only on the treatment effect at the discontinuation visit and not on treatment effects at earlier visits, and therefore $K_{t}$ has non-zero elements only in the final column; our software implementation below relies on this assumption. For tipping point sensitivity analyses, we consider two single-parameter causal models for the outcome at visit u after discontinuation at visit t:

(5) $$\;E\;\left[Y_{u}(t)-Y_{u}(0)\right]=k_{0}\;E\;\left[Y_{t}(t)-Y_{t}(0)\right]$$

(6) $$\;E\;\left[Y_{u}(t)-Y_{u}(0)\right]=k_{1}^{v_{u}-v_{t}}\;E\;\left[Y_{t}(t)-Y_{t}(0)\right]$$

where $v_{u},v_{t}$ are the times (on a suitable scale) of visits u,t. The maintained treatment effect after treatment discontinuation is constant in model (5) but decays exponentially in model (6), being multiplied by $k_{1}$ for every unit of time, where $0\leq k_{1}\leq 1$. A combined model is

(7) $$\;E\;\left[Y_{u}(t)-Y_{u}(0)\right]=k_{0}k_{1}^{v_{u}-v_{t}}\;E\;\left[Y_{t}(t)-Y_{t}(0)\right].$$

Next, we need to fix $\beta_{t}(t)$, the matrix of regression coefficients of $Y_{>t}(t)$ on $Y_{\leq t}(t)$. Assumptions A2 and A3 identify $\beta_{t}(0)$, the regression of $Y_{>t}(0)$ on $Y_{\leq t}(0)$, and $\beta_{t}(t_{max})$, the regression of $Y_{>t}(t_{max})$ on $Y_{\leq t}(t)$, respectively. We propose assuming either $\beta_{t}(t)=\beta_{t}(0)$ or $\beta_{t}(t)=\beta_{t}(t_{max})$. We call these “regression from reference” and “regression from active”, respectively. If all treatment effects are homogeneous (i.e. if Y(t)−Y(0) does not vary between individuals for any t) then $\beta_{t}(t)=\beta_{t}(t_{max})=\beta_{t}(0)$ and both “regression from reference” and “regression from active” are valid. If we are willing to assume equal variance-covariance matrices across trial arms ($\Sigma (t_{max})=\Sigma (0)$) then $\beta_{t}(t_{max})=\beta_{t}(0)$ and “regression from reference” and “regression from active” give the same results. The same arguments and proposals apply for $\Omega_{t}(t)$.

### 3.4. Comparison with reference-based imputation

From Table 1, RBI methods J2R, CIR and CR correspond to particular choices of the causal model, while the MAR and LMCF methods do not correspond to this causal model. $K_{t}$ is set to 0 for J2R, $C_{t}$ for CIR, and $\beta_{t}(0)$ for CR. This makes precise the statement of Mallinckrodt et al. (2012) that, under CIR, CR and J2R, the Z=a,D=t subgroup has the treatment effect at visit t maintained, diminished and eliminated, respectively, at visit $t_{max}$. Further, $\beta_{t}(t)$ and $\Omega_{t}(t)$ are set to $\beta_{t}(0)$ and $\Omega_{t}(0)$. If the RBI alternative variance structures are used then the same equivalences apply, but with $\beta_{t}(t)=\beta_{t}(t_{max})$ and $\Omega_{t}(t)=\Omega_{t}(t_{max})$.

## 4. Estimation

The CRK algorithm described in section 2 is easily adapted to impute under the causal model. Steps 1 and 2 are unchanged, and provide draws of μ(0), Σ(0), $\mu (t_{max})$ and $\Sigma (t_{max})$. Step 3 is skipped since the imputation distribution is directly derived from the causal model. Step 4 starts by imputing any missing data in the control arm under assumption A2, and any missing data in the active arm before treatment discontinuation under assumption A3. It then constructs the imputation distribution for active-arm data after treatment discontinuation using specification of $K_{t}$, $\beta_{t}(t)$ and $\Omega_{t}(t)$ as in Section 3.3. Steps 5 and 6 are unchanged.

We describe implementation using the SAS macros developed by James Roger to perform MI under the RBI methods. These are available on the web page (on www.missingdata.org.uk) of the DIA working group for missing data. We modified the Part2A macro to impute under the causal model with

(8) $$K_{t}=kC_{t}$$

where k is a scalar that may vary between participants. This enables causal model (5) to be implemented by setting $k=k_{0}$, the same for all participants. When interest is in the outcome at visit $t_{max}$, causal model (6) can be implemented by setting $k=k_{1}^{t_{max}-D}$, which varies across participants with different values of D. The modified macro is available on the DIA working group web page and sample code is provided in supplementary appendix C. By default, the variance-covariance matrices in the two arms, $\Sigma (t_{max})$ and Σ(0), are assumed equal, but the user can specify them to be unequal, which is the case we consider.

Alternative implementations are given in supplementary appendix D.

## 5. Simulation

We performed a simulation study to verify equivalence of the RBI methods with the proposed causal model for estimating the treatment effect at the final visit and to assess the impact of mis-specification of $K_{t}$ and $\beta_{t}(t)$. Mis-specification of $\Omega_{t}(t)$ has no impact on bias and little impact on variance, and for brevity is not discussed.

### 5.1. Design

Details of the data generating mechanism for simulating the observed and unobserved data are given in appendix E. Briefly, we consider an RCT with one baseline observation and two post-baseline visits during the treatment period (that is, $t_{max}=2$). Some active-arm participants discontinue treatment after visit 1 and are not observed at time 2; all other participants continue randomised treatment and are fully observed. The mechanism for discontinuing treatment is either MCAR or MAR. The data distribution for the observed data has either $\beta_{1}(2)=\beta_{1}(0)$ or $\beta_{1}(2)\neq \beta_{1}(0)$: the latter is designed to make choices of $\beta_{1}(1)$ important.

For each mechanism for simulating the observed data, we analysed the data in three ways, each with several different settings. For *complete data*, we generated the unobserved data using a maintained treatment effect parameter k=0,0.5,0.74 or 1 and setting $\beta_{1}(1)=\beta_{1}(0)$ or $\beta_{1}(2)$. For *causal model imputation*, we imputed the missing data using the causal model assuming a maintained treatment effect parameter $\tilde{k}=0,0.5,0.74$ or 1, and setting ${\tilde{\beta }}_{1}(1)$, the assumed value of $\beta_{1}(1)$, equal to $\beta_{1}(0)$ or $\beta_{1}(2)$. For *RBI imputation*, we imputed the missing data using the reference-based imputation methods CR, CIR and J2R with the variance-covariance matrix taken from the control arm or from the active arm. In all cases, we estimated the treatment effect from a linear regression of $Y_{2}$ on randomised arm and baseline $Y_{0}$. With imputed data, standard errors were computed using Rubin’s rules.

### 5.2. Results

Table 2 displays the average estimated treatment difference at visit 2 for each data generating mechanism (columns) and each analysis method (rows).

**Table 2.** Simulation study with D=1 or 2: estimates of treatment effect at visit 2 using complete data, causal model imputation and RBI imputation. $\beta_{1}(0)=(0,0.5{)}^{\prime }$ in all cases. $\beta_{1}(2)\neq \beta_{1}(0)$ means $\beta_{1}(2)=(-0.12,0.74{)}^{\prime }$.

	Data generating mechanisms for observed data			
	MCAR		MAR	
	$\beta_{1}(2)=\beta_{1}(0)$	$\beta_{1}(2)\neq \beta_{1}(0)$	$\beta_{1}(2)=\beta_{1}(0)$	$\beta_{1}(2)\neq \beta_{1}(0)$
**A**. Complete data generated with:				
$\beta_{1}(1)=\beta_{1}(0)$				
k=0.00	0.99	0.99	1.00	0.70
k=0.50	1.24	1.24	1.25	0.95
k=0.74	1.36	1.36	1.37	1.07
k=1.00	1.49	1.49	1.50	1.20
$\beta_{1}(1)=\beta_{1}(2)$				
k=0.00	0.99	1.00	1.00	1.00
k=0.50	1.24	1.25	1.25	1.25
k=0.74	1.36	1.37	1.37	1.37
k=1.00	1.49	1.50	1.50	1.50
**B**. Causal model imputation with assumed ${\tilde{\beta }}_{1}(1)$ and $\tilde{k}$				
${\tilde{\beta }}_{1}(1)=\beta_{1}(0)$				
$\;\tilde{k}=0.00$	1.00	1.00	1.00	0.71
$\;\tilde{k}=0.50$	1.24	1.25	1.25	0.96
$\;\tilde{k}=0.74$	1.36	1.37	1.37	1.08
$\;\tilde{k}=1.00$	1.49	1.50	1.50	1.21
${\tilde{\beta }}_{1}(1)=\beta_{1}(2)$				
$\;\tilde{k}=0.00$	1.00	1.00	1.00	1.00
$\;\tilde{k}=0.50$	1.24	1.25	1.25	1.25
$\;\tilde{k}=0.74$	1.36	1.37	1.37	1.37
$\;\tilde{k}=1.00$	1.49	1.50	1.50	1.50
**C**. RBI imputation with assumed variance-covariance and method				
Variance-covariance matrix from control arm				
J2R	1.00	1.00	1.00	0.71
CR	1.24	1.25	1.25	0.96
CIR	1.49	1.50	1.50	1.21
Variance-covariance matrix from active arm				
J2R	1.00	1.00	1.00	1.00
CR	1.24	1.37	1.25	1.38
CIR	1.49	1.50	1.50	1.50

Note: Maximum Monte Carlo standard error <0.01

Comparing panels A (analysis of complete data) and B (analysis by causal model imputation) shows that the causal model imputation methods result in unbiased estimates when the assumed values of ${\tilde{\beta }}_{1}(1)$ and $\tilde{k}$ agree with the true values of $\beta_{1}(1)$ and k.

Comparing panels B and C (analysis by RBI imputation) shows that the RBI estimates with variance-covariance matrix drawn from the control arm (as in CRK) agree with specific cases of the causal model estimates with $\beta_{1}(1)=\beta_{1}(0)$, and the RBI estimates with variance-covariance matrix drawn from the active arm (as in RBI alternative) agree with specific cases of the causal model estimates with $\beta_{1}(1)=\beta_{1}(2)$. Specifically, J2R corresponds to $\tilde{k}=0$, CIR corresponds to $\tilde{k}=1$, and CR corresponds to $\tilde{k}=$ the second element of $\beta_{1}(1)$ which is 0.50 or 0.74 depending on the data generating mechanism.

Comparing different choices of ${\tilde{\beta }}_{1}(1)$ when the observed data had either no selection effect (i.e. under MCAR) or $\beta_{1}(0)=\beta_{1}(2)$ (that is, in the first three data generating mechanisms), we see that choice of ${\tilde{\beta }}_{1}(1)$ does not affect estimates, as expected from Section 3.4. Sensitivity to choice of ${\tilde{\beta }}_{1}(1)$ was observed in the fourth data generating mechanism (MAR with $\beta_{1}(0)\neq \beta_{1}(2)$): mean causal model estimates were reduced by 0.29 by assuming $\beta_{1}=\beta_{1}(0)$ instead of $\beta_{1}=\beta_{1}(2)$. Sensitivity to choice of $\tilde{k}$ was the same for all values of $\beta_{1}$ (see Section 3.4): for example, assuming $\tilde{k}=0$ instead of $\tilde{k}=1$ reduced mean causal model estimates by 0.50 irrespective of the value of $\beta_{1}$.

Table 3 displays the average standard error (SE) (the average of the 1000 SEs) and the empirical SE (the sample standard deviation of the 1000 point estimates) for the treatment difference at the final visit. Empirical and average SEs for J2R and CIR are similar to those for the corresponding causal model estimates. The SEs for CR are slightly larger than those for the causal model with $\tilde{k}=$ the second element of $\beta_{1}$, because $\beta_{1}$ is estimated in CR while $\tilde{k}$ is an assumed value in the causal model. With MAR data, the larger average and empirical SEs due to using the variance-covariance matrix from the active arm rather than from the control arm arise mainly because there are no missing data in the control arm and heterogeneity is larger in the active arm than in the control arm. More importantly, as shown in Seaman et al. (2014), the results confirm that both RBI and causal model methods give (1) smaller empirical SEs than the estimator based on the complete data, and (2) larger average SEs (estimated using Rubin’s rules) than the empirical SEs of the methods and the empirical SEs based on the complete data. We comment on these observations in the discussion.

**Table 3.** Simulation study: average standard error (empirical standard error) for the treatment difference at the final visit using complete data, causal model imputation and RBI imputation. $\beta_{1}(0)$ and $\beta_{1}(2)$ as in Table 2.

	Data generating mechanisms for observed data			
	MCAR		MAR	
	$\beta_{1}(2)=\beta_{1}(0)$	$\beta_{1}(2)\neq \beta_{1}(0)$	$\beta_{1}(2)=\beta_{1}(0)$	$\beta_{1}(2)\neq \beta_{1}(0)$
**A**. Complete data generated with:				
$\beta_{1}(1)=\beta_{1}(0)$				
k=0.00	0.276 (0.273)	0.318 (0.311)	0.260 (0.255)	0.295 (0.288)
k=0.50	0.272 (0.269)	0.315 (0.308)	0.261 (0.256)	0.298 (0.291)
k=0.74	0.302 (0.206)	0.342 (0.247)	0.311 (0.219)	0.351 (0.258)
k=1.00	0.270 (0.266)	0.313 (0.306)	0.262 (0.257)	0.301 (0.295)
$\beta_{1}(1)=\beta_{1}(2)$				
k=0.00	0.276 (0.273)	0.318 (0.316)	0.260 (0.255)	0.292 (0.288)
k=0.50	0.272 (0.269)	0.315 (0.312)	0.261 (0.256)	0.296 (0.291)
k=0.74	0.271 (0.268)	0.314 (0.311)	0.262 (0.256)	0.298 (0.294)
k=1.00	0.270 (0.266)	0.313 (0.310)	0.262 (0.257)	0.300 (0.296)
**B**. Causal model imputation with assumed ${\tilde{\beta }}_{1}(1)$ and $\tilde{k}$				
${\tilde{\beta }}_{1}(1)=\beta_{1}(0)$				
$\;\tilde{k}=0.00$	0.310 (0.168)	0.337 (0.189)	0.305 (0.171)	0.328 (0.181)
$\;\tilde{k}=0.50$	0.301 (0.190)	0.327 (0.226)	0.299 (0.189)	0.322 (0.214)
$\;\tilde{k}=0.74$	0.302 (0.206)	0.327 (0.249)	0.301 (0.206)	0.325 (0.237)
$\;\tilde{k}=1.00$	0.305 (0.226)	0.332 (0.277)	0.306 (0.227)	0.332 (0.267)
${\tilde{\beta }}_{1}(1)=\beta_{1}(2)$				
$\;\tilde{k}=0.00$	0.310 (0.168)	0.359 (0.187)	0.315 (0.188)	0.359 (0.206)
$\;\tilde{k}=0.50$	0.301 (0.190)	0.344 (0.224)	0.310 (0.204)	0.350 (0.236)
$\;\tilde{k}=0.74$	0.302 (0.206)	0.342 (0.247)	0.311 (0.219)	0.351 (0.258)
$\;\tilde{k}=1.00$	0.305 (0.226)	0.345 (0.275)	0.316 (0.239)	0.356 (0.285)
**C**. RBI imputation with assumed variance-covariance matrix and method				
Variance-covariance matrix from control arm				
J2R	0.310 (0.168)	0.337 (0.189)	0.305 (0.171)	0.328 (0.181)
CR	0.303 (0.192)	0.328 (0.229)	0.306 (0.200)	0.331 (0.226)
CIR	0.305 (0.226)	0.332 (0.277)	0.306 (0.227)	0.332 (0.267)
Variance-covariance matrix from active arm				
J2R	0.310 (0.168)	0.359 (0.187)	0.315 (0.188)	0.359 (0.206)
CR	0.305 (0.192)	0.344 (0.249)	0.332 (0.236)	0.370 (0.283)
CIR	0.305 (0.226)	0.345 (0.275)	0.316 (0.239)	0.356 (0.285)

Note: Maximum Monte Carlo standard error <0.0005

## 6. Examples

We use two example data sets from randomised, double-blind, parallel-group studies comparing active treatment with placebo. The first is from a trial of 172 participants with major depressive disorders, taken from the DIA page of www.missingdata.org.uk, and used in the DIA working group to demonstrate various missing data related analytical methods. The outcome variable is the 17-item Hamilton Depression Rating Scale, HAMD17. The second, kindly supplied by Devan Mehrotra, is from a pain trial with a pain score as outcome.

**Figure 2.**
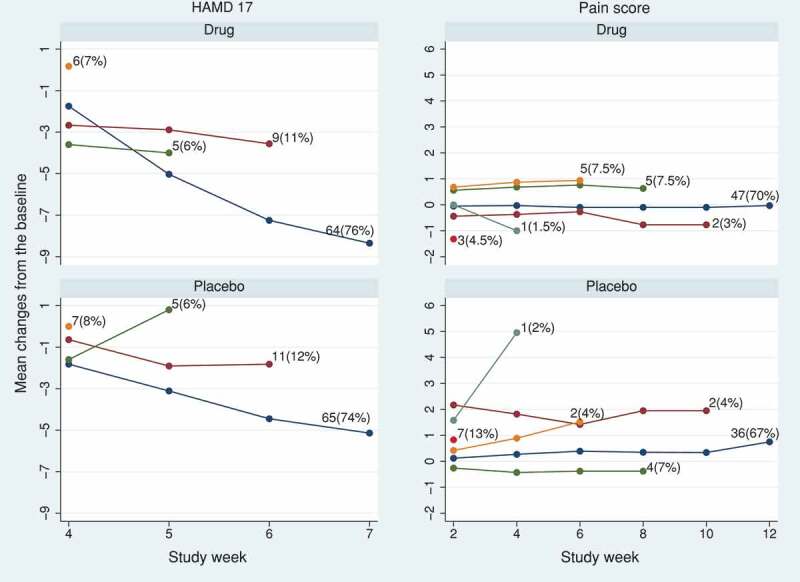
HAMD17 and pain score data sets: observed mean profile according to the visit at which treatment was discontinued in the active and placebo arms. Note: In the pain score data, four subjects in the active arm and two subjects in the placebo arm did not complete any post-baseline visit and were excluded from analysis.

In the HAMD17 trial, 76% (64/84) and 74% (65/88) of the randomised participants completed the final (fourth) visit in the active and placebo arms, respectively. In the pain score trial, the completion rate at the final (sixth) visit was 70% (47/67) and 67% (36/54) in the active and placebo arms, respectively. In both trials, participants were not followed up after treatment discontinuation. The observed trajectory means and the frequency of dropout patterns in each trial are shown in Figure 2.

We used the SAS 5 macros for implementing the RBI methods and causal models (Section 4). For the RBI methods, we assumed participants in the active arm were treated similarly to the placebo arm after discontinuing the active treatment. To construct the joint distribution of pre- and post-discontinuation active-arm data under the RBI methods, we first used the variance-covariance matrix from the placebo arm (RBI analyses) and then repeated the methods with the variance-covariance matrix from the active arm (RBI alternative analyses).

Table 4 shows the estimated treatment effect on HAMD17 and pain score at the final visit from standard MI, MMRM and RBI methods. The standard MI and MMRM methods estimate the *de jure* estimand. These differ slightly for HAMD17 because of a small incompatibility between the imputation and analysis models: the imputation model uses all visits to estimate a common effect of the baseline covariate PoolInv, but the analysis model uses only the final visit. The RBI methods estimate the *de facto* estimand and show, as expected, treatment estimates of smaller magnitude than the *de jure* estimand, with J2R giving the smallest magnitude of treatment effect followed by CR. Using the variance-covariance matrix from the active arm rather than from the placebo arm gives slightly more conservative estimates.

**Table 4.** HAMD17 and pain score data: estimated treatment effect at the final visit using standard multiple imputation with 100 imputations, mixed model for repeated measures (MMRM) and RBI methods.

*Estimand* &	HAMD17			Pain score		
Method	Estimate${\;}^{1}$	Std. error	*p*-value	Estimate${\;}^{2}$	Std. error	*p*-value
*De jure*						
Standard MI	−2.62	0.99	0.01	−0.88	0.39	0.03
MMRM	−2.58	1.03	0.01	−0.88	0.39	0.03
*De facto*						
RBI: variance-covariance matrix from the placebo arm						
J2R	−2.01	1.01	0.05	−0.64	0.40	0.11
CR	−2.22	0.99	0.03	−0.75	0.39	0.06
CIR	−2.30	0.99	0.02	−0.77	0.39	0.05
RBI alternative: variance-covariance matrix from the drug arm						
J2R	−1.99	1.01	0.05	−0.60	0.39	0.13
CR	−2.20	0.99	0.03	−0.71	0.39	0.07
CIR	−2.28	0.99	0.02	−0.73	0.39	0.06

${\;}^{1}$ Monte Carlo standard error for MI methods is ≤0.04.

${\;}^{2}$ Monte Carlo standard error for MI methods is ≤0.02.

**Figure 3.**
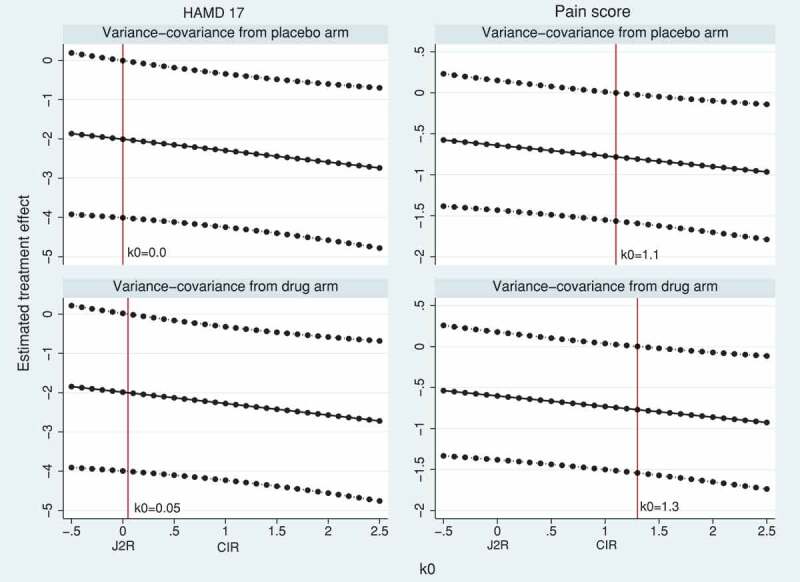
HAMD17 and pain score data sets: tipping point analysis for the estimated treatment effect at the final visit using causal model (5). The model has a constant treatment effect after treatment discontinuation, equal to fraction $k_{0}$ of the treatment effect at treatment discontinuation. The horizontal solid and dotted lines represent the treatment estimates and their pointwise 95% CI, respectively. The vertical solid line corresponds to $k_{0}$ such that *p*-value >0.05 in the left-hand side of the line (tipping point).

We next demonstrate tipping point sensitivity analyses using causal models (5) and (6). In model (5), a fraction $k_{0}$ of the treatment effect is maintained at all visits after discontinuation. Figure 3 shows the *de facto* estimates and 95% CI over a range of $k_{0}$ from −0.5 to 2.5. As shown in the theory and the simulation results, the J2R and CIR estimates correspond to using the causal model with $k_{0}=0$ (no maintained treatment effect after discontinuation) and $k_{0}=1$ (fully maintained treatment effect after discontinuation), respectively. Values $k_{0}<0$ mean that the effect of treatment after discontinuation is harmful, while values $k_{0}>1$ mean that the effect of treatment after discontinuation is greater than before discontinuation. The tipping point analysis on HAMD17 shows that statistical significance is lost when $k_{0}<0$ (with variance-covariance from the placebo arm) or $k_{0}<0.05$ (with variance-covariance from the active arm). In both cases, this suggests that the *de facto* estimate of treatment effect on HAMD17 is non-significant only if any benefit of the active treatment is lost immediately following discontinuation. For the pain score trial, statistical significance is lost when $k_{0}<1.1$ or $k_{0}<1.3$ (depending on whether the variance-covariance matrix is taken from the placebo or the active arm, respectively). This suggests that, in order for the *de facto* estimate of treatment effect to be statistically significant, there would need to be a delayed benefit such that the treatment effect was greater after discontinuation than before discontinuation. In both trials, comparing Table 4 with Figure 3 shows that the MAR analyses give estimates of the *de jure* estimand that are numerically similar to the causal model estimates of the *de facto* estimand when values around $k_{0}=2$ are assumed.

**Figure 4.**
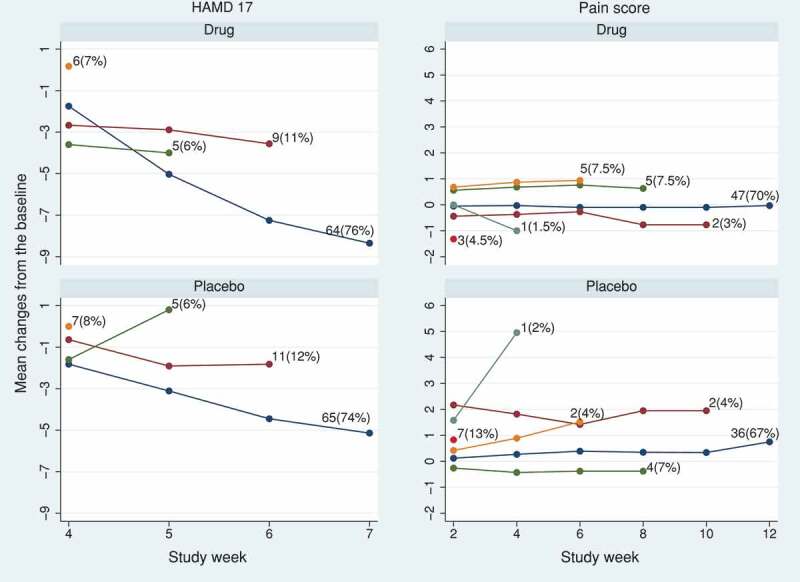
HAMD17 and pain score data sets: tipping point analysis for the estimated treatment effect at the final visit using causal model (6). The model has the treatment effect decaying exponentially after treatment discontinuation, by a ratio $k_{1}$ for each visit. The horizontal solid and dashed lines represent the treatment estimates and their pointwise 95% CI, respectively. The tipping point is not attained in the range $0\leq k_{1}\leq 1$.

In model (6), the treatment effect decays exponentially after discontinuation. Here, $k_{1}=0$ for J2R and 1 for CIR. We took visits as the timescale, so that $v_{t}=t$ in model (6). Figure 4 shows the *de facto* estimates of treatment effect at the final visit and its 95% CI from the causal model over a range of $k_{1}$. This model does not accommodate the effect of treatment after discontinuation being either harmful or greater than before discontinuation, and because of the more limited range of $k_{1}$, the tipping point is not reached: all results are statistically significant for HAMD17 and not significant for the pain score.

## 7. Discussion

We have considered longitudinal RCTs with quantitative outcomes in which participants who discontinue an active treatment are not followed up thereafter, but are assumed to receive a treatment similar to the control treatment. We have focused on estimating the effect of assignment to treatment in the actual treatment circumstances of the trial (*de facto* or treatment-policy estimand) rather than the treatment effect if all participants had tolerated or adhered to trial protocol (*de jure* or hypothetical estimand). We have proposed a generalised causal modelling approach to account for treatment discontinuation in the estimation of the *de facto* estimand. The proposed causal model makes an explicit assumption about the maintained causal effect of treatment after treatment discontinuation and provides flexibility to perform sensitivity analyses to the causal assumption. The causal model agrees with RBI methods in certain cases, and this provides a formal justification of these RBI methods.

The proposed causal model specifies how much of the treatment effect is maintained after treatment discontinuation, which we represent by the matrix $K_{t}$. We illustrated this with two examples of $K_{t}$: equation (5) with the maintained treatment effect independent of time since discontinuation, and equation (6) with the maintained treatment effect decaying exponentially with visits since discontinuation. A simple extension would allow $K_{t}$ to depend on the reason for treatment discontinuation. Ideally, sponsors should justify the choice of $K_{t}$ in the trial protocol based on the nature of the trial and the treatments.

The choice of regression slope $\beta_{t}(t)$ in the imputation model, reflecting within-subject dependence of post-discontinuation outcomes on pre-discontinuation outcomes should similarly be pre-specified. It is hard to recommend a single choice and perhaps both $\beta_{t}(t)=\beta_{t}(0)$ and $\beta_{t}(t)=\beta_{t}(t_{max})$ should be implemented. If the analyst is willing to assume equal variance-covariance matrices across trial arms then the situation is simpler and $\beta_{t}(t)=\beta_{t}(0)=\beta_{t}(t_{max})$ is the obvious choice. It is sensible to make the corresponding choices for the residual variance matrix $\Omega_{t}(t)$.

Our model has been presented for the case of active-arm treatment discontinuation, where subjects who discontinue do not then receive rescue medication over and above the per-protocol treatment regime for the control arm or when interest is in the effect attributable to the initially randomised treatment without the confounding effects of rescue medications. An unresolved problem is how to handle initiation of rescue medications when the confounding effects of rescue medications are of interest. The model can be extended to handle the control arm starting active treatment: an assumption like A6 still holds, but the $K_{t}$ matrix must be replaced by assumptions about how the treatment effect builds up over time.

Assumption A6 implies that if treatment has no effect before discontinuation then it has no effect after discontinuation. This seems reasonable in general; if it was unreasonable in a particular trial, then a constant term could be added in assumption A6 and equation (1). Other assumptions are possible, such as a non-linear model.

We have focussed on varying assumption A6, but we should also assess a number of other assumptions. The MAR assumptions A2 and A3, and the related assumption A5, could be made more plausible if the model could be extended to include further time-dependent covariates. Alternatively one could explore sensitivity to these assumptions by methods like those of Ratitch et al. (2013). It is less clear how to assess departures from the linearity assumption A4.

All the methods we have considered – RBI methods, causal model and MMRM – make a multivariate Normal (MVN) assumption. Our key finding that the causal model and RBI methods are equivalent is valid even if the MVN assumption is false. However, failure of the MVN assumption risks causing bias in all the methods. The assumption can be checked in the observed data using standard methods. If the MVN assumption is correct for the *observed* data and the maintained treatment effect model (6) is correct, then the imputed data have the correct mean, and so the treatment effect in the imputed data is unbiased even if the MVN assumption is false for the *unobserved* data. If data were skewed, then it would be wise to consider a transformation before analysis.

Our model applies to quantitative outcomes. Extension to other outcomes would be useful.

The repeated-sampling variance of the estimated treatment effect tends to be smaller than the Rubin’s rules estimate of variance for a given $K_{t}$ (Table 3). The repeated-sampling variance can be approximated in practice using the delta method (Liu and Pang 2016; Oehlert 1992). Carpenter et al. (2014) argue that the repeated-sampling variance is not appropriate since it is typically smaller than the complete-data variance (to an extent which depends on the value of $K_{t}$). They also argue that the Rubin’s rules estimate of variance of the treatment effect is larger than the complete-data variance, because of the information lost due to the missing data, and this makes it an appropriate variance (Carpenter et al. 2014; Cro et al. 2019). We point out that the type I error rate is correct for the repeated-sampling variance and too small for the Rubin’s rules variance, meaning that the Rubin’s rules variance carries a loss of power; therefore, the repeated-sampling variance may be appropriate for a primary analysis.

In summary, whilst MI is an attractive and powerful method for handling missing data in both experimental and observational studies, it is not always clear what estimand is being targeted or what assumptions are being made about how outcomes for subjects who discontinue randomised treatment relate to those who remain on study. The recent estimands debate (European Medicines Agency 2017) has led to a growing recognition that more complex estimation approaches that do not rely on randomisation may be needed to handle post-randomisation events that lead to missing data, and there are calls for causal inference methods to become more widely adopted (e.g. Akacha et al. (2017); Little and Kang (2015)). We join this call to encourage greater understanding and application of ideas from the causal inference literature to help support the definition and estimation of estimands of interest in a randomised clinical trial. We hope that this paper illustrates how a causal inference framework can provide clarity and rigour in stating estimands, stating assumptions, and performing estimation.

## References

[CIT0001] Akacha, M., F. Bretz, and S. Ruberg. 2017. Estimands in clinical trials - broadening the perspective. *Statistics in Medicine* 36 (1):5–19. doi:10.1002/sim.v36.1. 27435045

[CIT0002] Angrist, J. D., G. W. Imbens, and D. B. Rubin. 1996. Identification of causal effects using instrumental variables. *Journal of the American Statistical Association* 91:444–455. doi:10.1080/01621459.1996.10476902.

[CIT0003] Carpenter, J. R., J. H. Roger, S. Cro, and M. G. Kenward. 2014. Response to comments by Seaman et al. on “analysis of longitudinal trials with protocol deviation: A framework for relevant, accessible assumptions, and inference via multiple imputation. *Journal of Biopharmaceutical Statistics* 24 (6):1363–1369. doi:10.1080/10543406.2014.960085. 25215553

[CIT0004] Carpenter, J. R., J. H. Roger, and M. G. Kenward. 2013. Analysis of longitudinal trials with protocol deviation: A framework for relevant, accessible assumptions, and inference via multiple imputation. *Journal of Biopharmaceutical Statistics* 23 (3):1352–1371. doi:10.1080/10543406.2013.834911. 24138436

[CIT0005] Cro, S., J. R. Carpenter, and M. G. Kenward. 2019. Information-anchored sensitivity analysis: Theory and application. *Journal of the Royal Statistical Society: Series A (Statistics in society)* 182 (2):623–645. doi:10.1111/rssa.2019.182.issue-2. PMC6378615 30828138

[CIT0006] Dunn, G., M. Maracy, C. Dowrick, J. L. Ayuso-Mateos, O. S. Dalgard, H. Page, V. Lehtinen, P. Casey, C. Wilkinson, J. L. Vazquez-Barquero, et al. 2003. Estimating psychological treatment effects from a randomised controlled trial with both non-compliance and loss to follow-up. *British Journal of Psychiatry* 183 (4):323–331. doi:10.1192/bjp.183.4.323. 14519610

[CIT0007] European Medicines Agency. 2017. ICH E9 (R1) addendum on estimands and sensitivity analysis in clinical trials to the guideline on statistical principles for clinical trials. Accessed 424, 2018. http://www.ema.europa.eu/docs/en_GB/document_library/Scientific_guideline/2017/08/WC500233916.pdf.

[CIT0008] Kenward, M., E. Goetghebeur, and G. Molenberghs. 2001. Sensitivity analysis for incomplete categorical data. *Statistical Modelling* 1 (1):31–48. doi:10.1177/1471082X0100100104.

[CIT0009] Little, R., and S. Kang. 2015. Intention-to-treat analysis with treatment discontinuation and missing data in clinical trials. *Statistics in Medicine* 34 (16):2381–2390. doi:10.1002/sim.v34.16. 25363683

[CIT0010] Little, R., and L. Yau. 1996. Intent-to-treat analysis for longitudinal studies with drop-outs. *Biometrics* 52 (4):1324–1333. doi:10.2307/2532847. 8962456

[CIT0011] Little, R. J., and D. B. Rubin. 2000. Causal effects in clinical and epidemiological studies via potential outcomes: concepts and analytical approaches. *Annual Review of Public Health* 21 (1):121–145. doi:10.1146/annurev.publhealth.21.1.121. 10884949

[CIT0012] Liu, G. F., and L. Pang. 2016. On analysis of longitudinal clinical trials with missing data using reference-based imputation. *Journal of Biopharmaceutical Statistics* 26 (5):924–936. doi:10.1080/10543406.2015.1094810. 26418282

[CIT0013] Liublinska, V., and D. B. Rubin. 2014. Sensitivity analysis for a partially missing binary outcome in a two-arm randomized clinical trial. *Statistics in Medicine* 33 (24):4170–4185. doi:10.1002/sim.6197. 24845086 PMC4297215

[CIT0014] Mallinckrodt, C. H., Q. Lin, I. Lipkovich, and G. Molenberghs. 2012. A structured approach to choosing estimands and estimators in longitudinal clinical trials. *Pharmaceutical Statistics* 11 (6):456–461. doi:10.1002/pst.v11.6. 22962024

[CIT0015] National Research Council. 2010. *The prevention and treatment of missing data in clinical trials*. Washington, DC: Panel on Handling Missing Data in Clinical Trials. Committee on National Statistics, Division of Behavioral and Social Sciences and Education. The National Academies Press.

[CIT0016] Oehlert, G. W. 1992. A note on the delta method. *The American Statistician* 46 (1):27–29.

[CIT0017] Permutt, T. 2016. A taxonomy of estimands for regulatory clinical trials with discontinuations. *Statistics in Medicine* 35 (17):2865–2875. doi:10.1002/sim.v35.17. 26678026

[CIT0018] Ratitch, B., M. O’Kelly, and R. Tosiello. 2013. Missing data in clinical trials: From clinical assumptions to statistical analysis using pattern mixture models. *Pharmaceutical Statistics* 12:337–347. doi:10.1002/pst.v12.6. 23292975

[CIT0019] Rubin, D. B. 1987. *Multiple imputation for nonresponse in surveys*. New York: John Wiley and Sons.

[CIT0020] Seaman, S., F. Leacy, and I. R. White. 2014. Re: Analysis of longitudinal trials with protocol deviations — a framework for relevant, accessible assumptions, and inference via multiple imputation (carpenter, Roger and Kenward, journal of biopharmaceutical statistics 2013;23:1352–1371). *Journal of Biopharmaceutical Statistics* 24:1358–1362. doi:10.1080/10543406.2014.928306. 24915418 PMC4241629

[CIT0021] Yan, X., S. Lee, and N. Li. 2009. Missing data handling methods in medical device clinical trials. *Journal of Biopharmaceutical Statistics* 19 (6):1085–1098. doi:10.1080/10543400903243009. 20183466

